# Conformational Temperature-Dependent Behavior of a Histone H2AX: A Coarse-Grained Monte Carlo Approach Via Knowledge-Based Interaction Potentials

**DOI:** 10.1371/journal.pone.0032075

**Published:** 2012-03-19

**Authors:** Miriam Fritsche, Ras B. Pandey, Barry L. Farmer, Dieter W. Heermann

**Affiliations:** 1 Institute for Theoretical Physics, University of Heidelberg, Heidelberg, Germany; 2 Department of Physics and Astronomy, University of Southern Mississippi, Hattiesburg, Mississippi, United States of America; 3 Materials and Manufacturing Directorate, Air Force Research Laboratory, Wright Patterson Air Force Base, Ohio, United States of America; 4 Institute for Molecular Biophysics, The Jackson Laboratory, Bar Harbor, Maine, United States of America; 5 Interdisciplinary Center for Scientific Computing, Heidelberg, Germany; University of Akron, United States of America

## Abstract

Histone proteins are not only important due to their vital role in cellular processes such as DNA compaction, replication and repair but also show intriguing structural properties that might be exploited for bioengineering purposes such as the development of nano-materials. Based on their biological and technological implications, it is interesting to investigate the structural properties of proteins as a function of temperature. In this work, we study the spatial response dynamics of the histone H2AX, consisting of 143 residues, by a coarse-grained bond fluctuating model for a broad range of normalized temperatures. A knowledge-based interaction matrix is used as input for the residue-residue Lennard-Jones potential.

We find a variety of equilibrium structures including global globular configurations at low normalized temperature (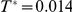), combination of segmental globules and elongated chains (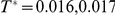), predominantly elongated chains (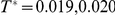), as well as universal SAW conformations at high normalized temperature (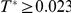). The radius of gyration of the protein exhibits a non-monotonic temperature dependence with a maximum at a characteristic temperature (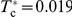) where a crossover occurs from a positive (stretching at 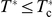) to negative (contraction at 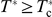) thermal response on increasing 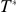.

## Introduction

Our perspective of proteins is more and more changing from static to dynamical entities [1]. Increasing efforts are undertaken to better understand the functioning of biological macromolecules with the aim of engineering them for technological applications [1]. In fact, proteins are powerful therapeutic agents [2], [3] and recent developments have stressed their impact on the bioengineering of nanomaterials, especially in biomedical imaging, drug delivery, biosensing and the design of functional nanocomposites [4], [5]. To this end, protein self-assembly in suitable media offers unique advantages in the fabrication of protein-based nanodevices and avoids cost-intensive manufacturing processes [1]. However, thermally stable proteins are essential for bionanotechnological applications [6]–[8]. Thus, based on the biological [9]–[12] and technological implications [1], [13], [14], it is interesting to investigate the structural properties of proteins as a function of temperature [15], [16].

Due to the unique sequence of residues a complex and specific structural response is expected. Consequently, the study of the structure of proteins has been of interest for many years, particularly exploiting computer simulations via idealized models [6], [7], [17]–[28]. However, despite considerable effort, a complete understanding of the fundamental issue of how proteins fold to its native structure still remains an open question from a first principle approach [6], [7], [17]–[28].

In this work, we explore how the protein H2AX, a variant of the histone H2A, conforms and responds to temperature changes. The protein H2AX plays a vital role in cellular functioning: Its serine 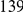 phosphorylated form, 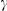-H2AX [29], is one of the earliest repair responses to DNA double-strand breaks which can lead to mutations that in turn are a cause of cancer and hereditary disease [30]. Notably, it was found that 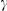-H2AX increases in a variety of conditions in relation to double-strand break generation processes, including radiation and high temperature [31]. Moreover, a recent work has shown that postranslational modifications in histones underlie heat acclimation-mediated cytoprotective memory [32].

Regarding the above mentioned technological implications of proteins in general and the biological impact of the histone H2AX in particular the study of their temperature-dependent structural changes is worthwhile. Thus, we consider a coarse-grained model [18], [20] of this protein chain where knowledge-based residue-residue interactions [19], [33] are employed and effects of temperature are explored.

## Analysis

The high dimensional space of protein conformations as well as the complexity of the energy surface make coarse-graining almost unavoidable in modeling the global structure and dynamics of proteins no matter whether one choses an all-atom approach, a minimalist description or a combination thereof [22]–[28].

In this work, we present a computer simulation study of the protein H2AX applying the bond-fluctuation method [34], which has been applied successfully to model the static and dynamical properties of polymer systems in several investigations [35]. It is a lattice algorithm with the advantage of avoiding non-ergodicity and its computational efficiency renders it more attractive than off-lattice models. The polymer evolves on a cubic lattice, where each monomer blocks eight lattice sites, which then cannot be occupied by other monomers. Monomers are connected by fluctuating bond vectors of lengths 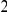, 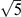, 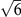, 3 and 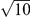, leading to an average bond length of 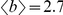. A Monte Carlo move consists of randomly choosing one monomer to be moved to a randomly chosen lattice direction by one lattice unit. This trial motion is only accepted if neither the excluded volume constraint nor the restriction on the allowed range of bond lengths is violated. By additionally considering the Metropolis transition probability [35] for accepting or rejecting a move we include effects due to the finite interaction energies explained in the following [34]. Thus, the simulation method produces unbiased results, takes into account excluded volume interactions and ensures that no bond crossings can occur.

The H2AX protein consists of 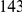 residues shown in Table 1. Each residue is described by a monomer of the bond-fluctuating protein chain [18], [20]. This is a simplified representation of a residue without the all-atom structural details but the specificity of each residue is captured via the applied residue-residue interactions [21]. Moreover, our approach has the advantage of computational efficiency allowing for the covering large (biological) scales. In fact, the bond-fluctuation method has recently been used to study the conformational relaxation into native structure of a general HP protein chain [36] and even a specific protein, sensory rhodopsin, without severe constraints [37].

**Table 1:** Sequence of residues of the histone H2AX.

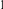 M	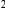 S	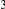 G	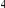 R	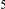 G	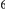 K	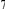 T	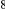 G	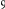 G	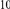 K	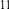 A	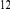 R
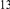 A	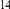 K	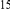 A	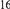 K	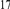 S	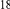 R	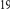 S	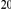 S	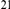 R	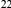 A	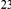 G	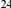 l
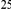 Q	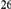 F	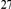 P	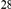 V	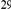 G	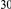 R	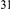 V	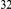 H	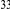 R	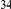 L	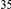 L	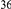 R
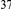 K	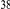 G	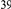 H	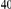 Y	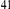 A	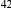 E	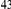 R	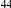 V	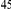 G	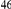 A	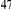 G	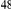 A
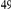 P	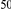 V	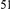 Y	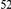 L	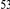 A	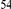 A	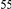 V	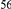 L	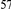 E	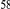 Y	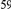 L	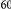 T
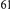 A	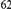 E	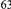 I	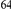 L	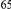 E	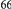 L	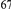 A	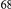 G	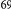 N	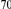 A	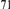 A	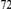 R
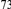 D	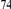 N	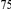 K	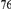 K	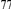 T	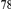 R	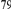 I	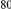 I	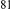 P	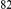 R	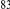 H	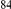 L
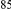 Q	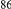 L	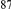 A	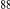 I	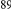 R	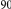 N	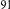 D	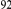 E	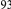 E	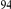 L	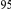 N	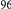 K
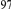 L	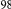 L	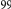 G	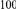 G	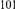 V	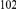 T	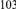 I	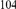 A	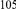 Q	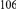 G	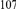 G	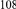 V
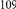 L	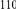 P	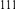 N	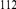 I	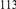 Q	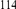 A	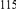 V	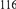 L	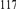 L	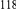 P	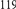 K	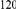 K
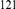 T	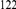 S	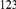 A	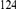 T	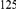 V	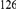 G	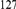 P	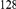 K	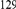 A	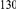 P	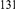 S	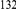 G
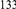 G	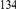 K	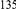 K	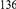 A	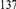 T	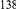 Q	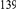 A	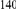 S	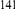 Q	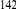 E	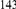 Y	

Hydrophobic residues are pink, polar residues are gold and electrostatic ones are blue.

Apart from excluded volume interactions, each residue interacts with the neighboring residues within the range 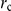 using a generalized Lennard-Jones (LJ) potential
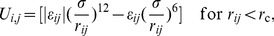
(1)where 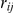 is the distance between the residues 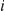 and 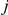 and 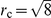 and 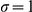 in units of the lattice constant. The strength of the pair potential 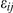 is unique for each pair of residues with appropriate positive (repulsive) or negative (attractive) values (for more detail on the force field see [19], [38]). In contrast to our recent study of a HIV protease with a coarse-grained approach involving the relative hydropathy index of each amino acid as well as results from all-atom simulations [18], in this work, we use a knowledge-based interaction matrix for the residue-residue pair interactions. The knowledge-based interaction potential matrix is derived from an ensemble of a large number of protein structures in the protein data bank (PDB). A number of such interaction tables are frequently used to investigate a range of questions related to protein structure including protein folding which has been studied extensively with a variety of models and methods involving all-atom details to minimalist coarse-grained descriptions [22]–[28], [39]–[44]. We resort here to the classic residue-residue contact interaction table [19] which is employed in studying scaffolding of short peptides [20].

Even though the knowledge-based matrix elements 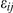 are simplified estimates derived from residue-residue contacts we are confident that the phenomenological interaction matrix implicitly takes into account the secondary and tertiary structure of the proteins [45], [46]. In fact, several factors need to be considered for the crystallization of a protein sample subject to an X-ray christallograpy study, which include protein purity, pH, concentration of the protein, the temperature, and precipitants. X-ray crystallographic images of several thousands of proteins from the PDB are used to derive the residue-residue interaction matrix which is applied as an input for our potential. In such a huge ensemble of proteins, residues in secondary and tertiary structures are expected be well represented by effective residue-residue interactions at various temperatures when applying a coarse-grained protein model.

Each randomly selected residue performs its stochastic movement according to the Metropolis algorithm subject to excluded volume constraints and the limits on changes in the covalent (i.e. peptide) bond length as in our previous studies [18], [20]. A randomly selected residue at a site 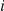 is moved to one of its randomly selected neighboring lattice sites 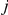 with the Boltzmann probability 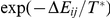, where 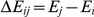 is the change in energy between the attempted 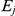 and current 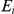 configuration [35].


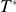
 is the normalized temperature in units of the Boltzmann constant 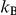 and the energy 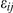. Due to the lack of calibration with experimental data it is not possible to quantitatively relate the temperature 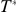 to physical temperature values. However, since the interresidue contact energies 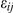 allow for the calculation of realistic conformational energies of amino acids sequences in a number of different folds [38] we are able to relate temperature changes qualitatively to changes in the structural properties of proteins. Thus, our coarse-grained protein model provides a (qualitative) framework for understanding the temperature-dependent response of proteins which can so far not be gained by experimental testing.

Initially, the protein chain is placed in a random conformation with excluded volume constraints. Simulations are then performed for a sufficiently long time (typically 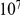 time steps) with 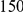 independent samples. While one can monitor (thermodynamic) quantities (such as the radius of gyration or the energy) in a simulation in order to make sure that the system has reached asymptotic steady state, one has to take into account that the protein may or may not be in equilibrium due to the possibility of metastability (caused by frustration). Gerstman and Chapagain [47] provide an estimate for the time which is required for a protein to undergo the transition from a random coil to its native state. Using a simplified coarse-grained model and introducing a propensity energy to constrain appropriate segmental structures they suggest that 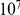 time steps (corresponding to about 0.01 sec) is large enough for a protein to reach its native structure. In our simulation, the protein chain is initially in a random coil configuration (with excluded volume constraints) and it takes about 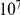 time steps to reach an equilibrium conformation. Thus, while one has to take into account that this approximation might fail when considering additional details such as an effective medium etc., the time scale by Gerstman and Chapagain could be a rough estimate of the order of magnitude for our simulation. Different lattice sizes are used to test for finite size effects. Most of the data presented here are generated on a lattice of size 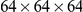 since the qualitative results for different lattice sizes do not show significant differences.

## Results

**Figure 1:**
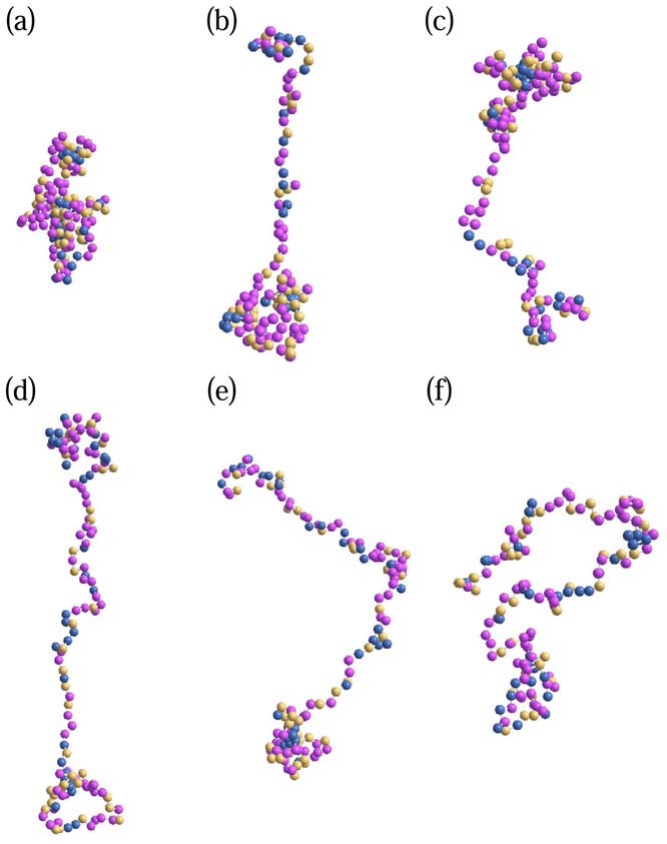
Snap shots of the histone H2AX at (a) 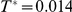, (b) 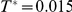, (c) 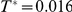, (d) 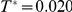, (e) 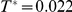 and (f) 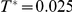. Hydrophobic residues are shown in pink, polar residues in gold and electrostatic ones in blue. We find globular configurations at low temperature (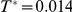), combination of segmental globules and elongated chains (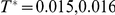), predominantly elongated chains (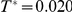), as well as universal SAW conformations at high temperature (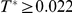).

Fig. 1 illustrates snapshots of the histone H2AX for different representative temperatures (in reduced units) in the range of 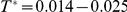. Some of the general conformational characteristics such as globular structure formation (global aggregation of the intra-chain residues, 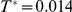), local segregation of selective residues (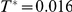), large-scale stretching (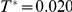), onset of randomization (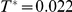) and thermal mixing (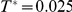) are already apparent in the “snapshot” configurations. The interplay between the cooperative and competing interactions among the residues and the temperature constrained by the peptide bonds leads to a rich ensemble of protein structures. While a detailed analysis of such a structural ensemble in an in vivo system still remains an open challenge, our approach offers some insights into the overall structural pattern changes that are so far inaccessible to experiment. Moreover, simulation studies (such as ours) preceding experimental tests may help e.g. in assessing the applicability of proteins for the design and fabrication of biomolecular devices in the bionanotechnology.

**Figure 2:**
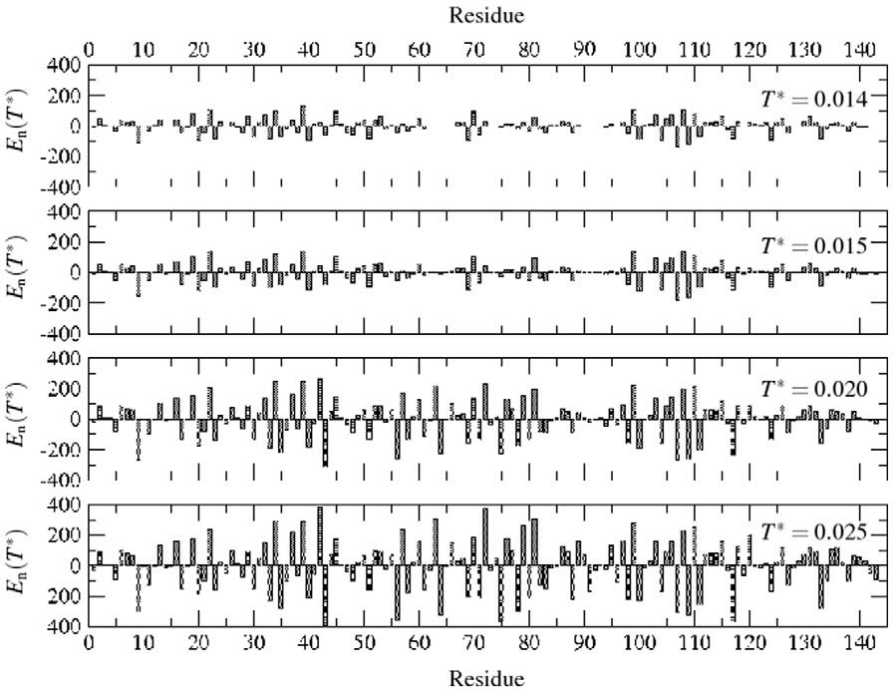
Energy 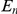 of each residue of histone H2AX at normalized temperatures 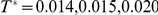, and 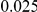. The energy of a residue is its interaction energy with neighboring residues within the range of interaction. The magnitude of the repulsive and the attractive energy and their differences in consecutive segments increases with temperature which is manifested in the global (coil-to-globule) structure of the protein. At low temperatures (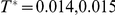), residues with the lowest mobility consist of 36R, 37K, 57E, 62E, 65E, 72R, 73D, 75K, 76K, 78R, 89R, 90N, 91D, 92E, 93E, 95N, 96K, 119K, 120K, 134K, 135K, 141Q and 142E. Nearly all the electrostatic residues (D, E, K, R) along with a few polar groups (Q,N) act as anchor/seed for segmental aggregation.

**Figure 3:**
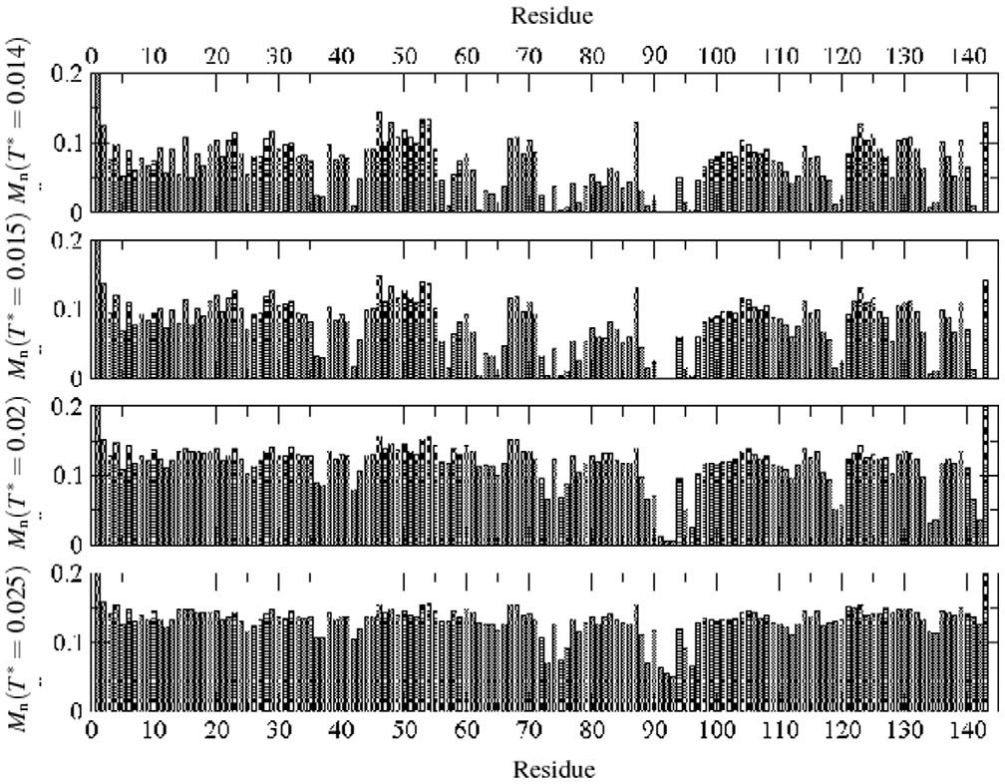
Mobility 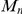 of each residue (number of successful moves per unit time step) of the histone H2AX at temperatures 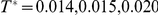, and 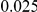. The mobility of a residue is defined by the number of successful moves per unit MC time step. With exceptions the mobility profile of the residues follows the energy profile relatively closely where the lower segmental energy differences translate into lower segmental mobility. Interestingly, some residues, 42E and 57E, show a low mobility despite their positive energy.

Fig. 2 and Fig. 3 show the energy and mobility profile of each residue. The energy of a residue is its interaction energy with neighboring residues within the range of interaction. The mobility of a residue is defined by the number of successful moves per unit MC time step. Note the contrasts in profiles at relatively low (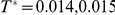) and high (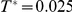) temperature. Residues along the histone backbone appear to possess an isotropic distribution of (almost in equal number) attractive (cohesive, negative) and repulsive (positive) energy. The magngitude of the repulsive and the attractive energy and their differences in consecutive segments increases with temperature which is manifested in the segmental configuration as well as in the global (coil-to-globule) structure of the protein.

The mobility profile of the residues follows the energy profile relatively closely where the lower segmental energy differences translate into lower segmental mobility. At low temperatures (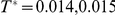), residues with the lowest mobility consist of 36R, 37K, 57E, 62E, 65E, 72R, 73D, 75K, 76K, 78R, 89R, 90N, 91D, 92E, 93E, 95N, 96K, 119K, 120K, 134K, 135K, 141Q and 142E. Nearly all the electrostatic residues (D, E, K, R) along with a few polar groups (Q,N) act as anchor/seed for segmental aggregation. Note that the pair interaction potentials of these residues have the largest well depth [19]. Most of these residues become more mobile when raising the temperature (i.e. see the segments 91D, 92E, 93E).

It should be pointed out that some residues (e.g. 42E and 57E) have surprisingly low mobility despite their positive energy while others with low energy have a high mobility index. In fact, conformational energy of the amino acids sequence (the interaction energy) does not determine the local structure and mobility alone. Physical (covalent bonding) or topological (trapping) constraints also play an important role in the cooperative response.

**Figure 4:**
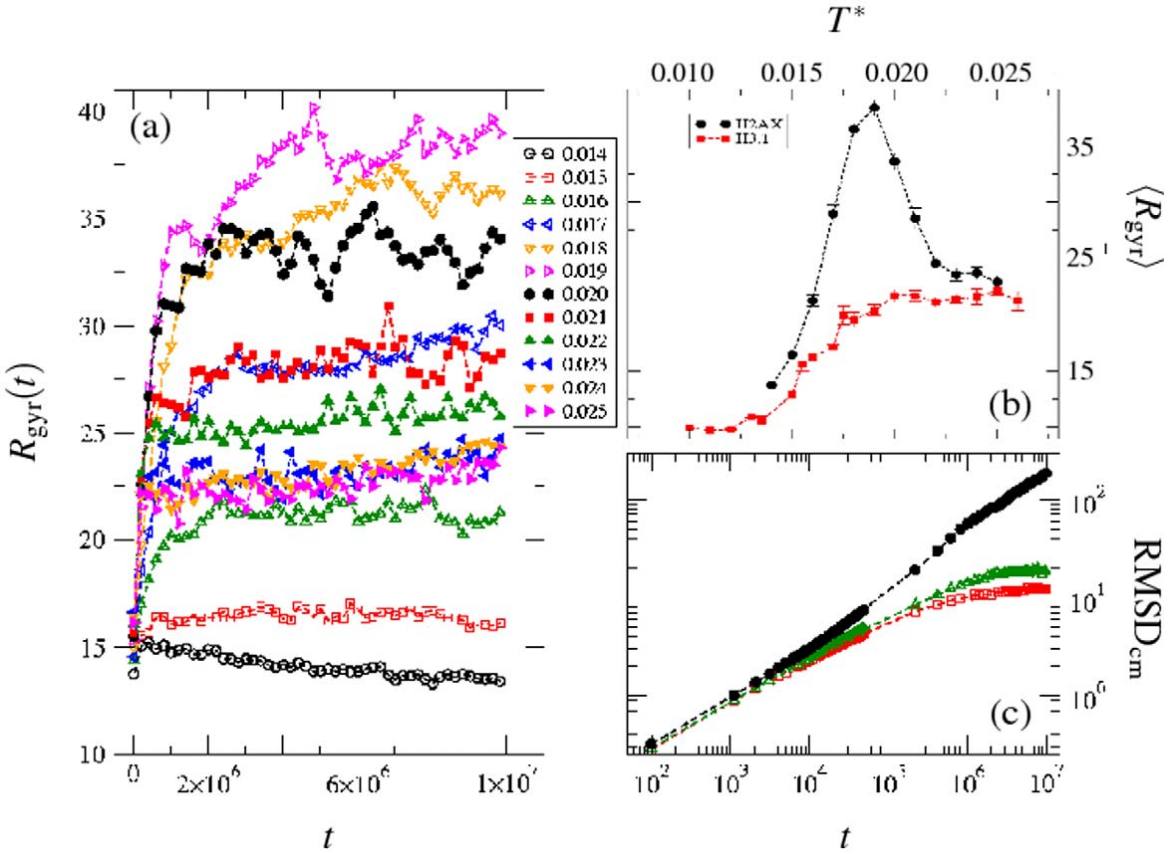
(a) Dependence of the radius of gyration 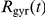 on MC time steps 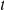 for various temperatures 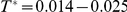; (b) temperature-dependent behavior of the mean radius of gyration 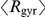 of the protein H2AX compared to the protein H3.1 where the same interaction parameters and normalized simulation temperature have been applied; (c) root mean squared displacement of the protein's center of mass 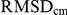 as a function of MC time steps 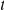. The crossover from a positive thermal response of 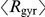 at low temperature to a negative thermal response at high temperature with a well-defined transition temperature 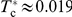 appears to be a specific characteristic of the H2AX protein structure.

With respect to biotechnological applications it is interesting to study how the entire range of temperatures affects a protein's size and shape. The temporal variation of the radius of gyration 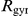 shows that it has reached its equilibrium at all temperatures except for the lowest one (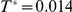) where the relaxation is too slow. Equilibration implies that the protein chain has explored a sufficient amount of conformations in structural phase space. The average value of the equilibrium radius of gyration can be evaluated from the asymptotic data sets at each temperature. 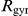 shows a non-monotonic dependence on temperature as can be seen in Fig. 4 with a maximum at a characteristic temperature 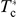 which is a “unique” property of the studied biomaterial. The radius of gyration increases on increasing the temperature (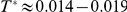) from the low end until around 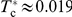 followed by a linear decay (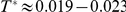) before reaching its saturation at high temperature 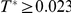.

We examine how the competition between residue-residue interactions and thermal fluctuations leads to the observed non-monotonic temperature dependence of the radius of gyration. The attractive inter-residue interaction induces self-assembly of the protein segments towards a global globular structure (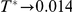) as the residues undergo their stochastic motion. As discussed above the highly interacting electrostatic residues act as an anchor collecting even those tethered residues that are repulsive. Thus, cooling down the protein leads to a conformational collapse into its globular conformation. The self-organized protein structure in its globular conformation begins to break on raising the temperature even by a small amount (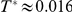) as the constitutive residues dissociate while some local assembly (held together by non-covalent interactions) still persists. The local assembly de-segregates on further increasing the temperature (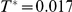) which stretches the corresponding segments resulting in a larger radius of gyration. Stretching of the protein continues until the characteristic temperature (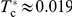) is reached beyond which the protein chain begins to contract. The chain segments fluctuate introducing randomness into a relatively stable elongated structure as the onset of thermal fluctuations sets in. The process can be described as a “thermal-driven contraction” emerging due to the cooperative effect of segmental interaction (looping) and conformational entropy. Note that the protein H2AX expands (positive) on heating in the low temperature regime (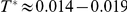) and contracts (negative) at the higher temperatures (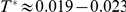).

The crossover from a positive thermal response in low temperature to a negative thermal response in high temperature regimes with a well-defined transition temperature (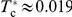) appears to be a specific characteristic of the H2AX protein structure. The results of another protein from histone family H3.1 provides additional support to our finding that histone H2AX shows a unique peak in its 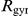-temperature profile. The same interaction parameters and normalized simulation temperature are applied in the study of H3.1, which is of comparable size and does not show any peak in its 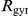-temperature profile but exhibits a continuous transition from coil-to-globule on reducing the temperature. The difference in thermal response of H2AX and H3.1 however leads us to believe that this is due to the specific sequence of amino acids which might be exploited in technological applications requiring a material with such a distinct temperature response. Eventually, the radius of gyration saturates on further increasing the temperature beyond 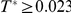, where the protein conforms to a thermal-driven random (coil) structure.

The size of the protein as measured by its radius of gyration 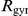 can be compared at a low temperature (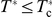) in the positive thermal response regime and at a higher temperature (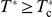) in the negative thermal response regime. One has to point out that, despite having the same magnitude of 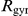, the structure of the protein at these temperatures is very different. In particular, as shown in Fig. 1 we observe a local segmental segregation at low temperature while random configuration at high temperature dominate.

Although the radius of gyration can provide insight into the spatial extension of the protein, the specific dynamics of local structures are difficult to quantify with this measure. Thus, we have analyzed the root mean squared displacement of the center of mass of the protein with as a function of time for the entire temperature range. Fig. 4 shows these results for representative temperatures. The protein continues to diffuse at high temperatures while its motion slows down on reducing the temperature showing sub-diffusive asymptotic dynamics. At very low temperatures, 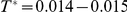, the dynamics are too slow since the protein is localized into its globular conformation.

**Figure 5:**
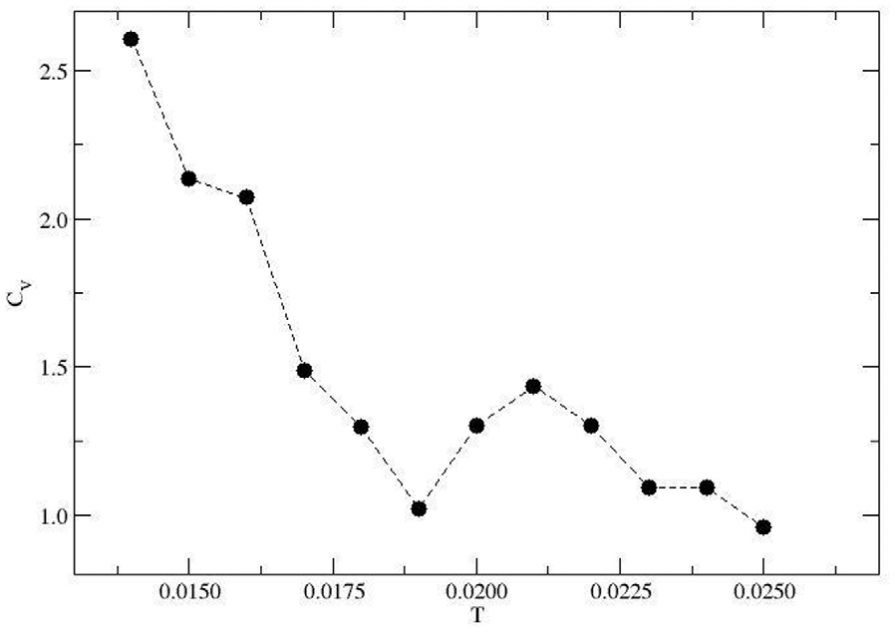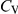 is evaluated from the fluctuation in the energy 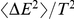 and shows a minimum in 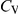 around 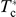. In fact, the relaxation of the protein is not only controlled by the competition between residue-residue interactions and temperature but also by the steric constraints imposed by the peptide bonds.

The question has to be raised whether there is another property such as the specific heat 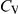 for which 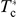 has a special significance. 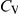 is evaluated from the fluctuation in the energy 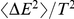 and Fig. 5 shows that the specific heat 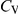 does not show a peak characteristic per se. This can be understood by noting that the characteristic temperature 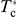 is related to the maximum thermal response in the spatial extension and not to the identification of a phase transition. However, we see that 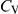 decays rather fast with increasing temperature before reaching a weave-like saturation. The undershoot in 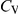 occurs around 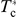 followed by an overshoot (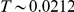) which could be seen as a minimum and maximum, respectively, in the temperature profile of 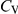. The thermal phase transition in general is associated with the divergence of the thermal correlation length at the critical point. The relaxation of the protein here is not only controlled by the competition between residue-residue interactions and temperature (i.e. the thermal fluctuation) but also by the steric constraints imposed by the peptide bonds (entropic barrier which is hard to evaluate). Moreover, the size of the protein is too small to identify the second order phase transition generally identified in macroscopically large (i.e. infinitely long ideal) chain systems. Thus, the thermal response in 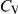 is expected to be different from that of the radius of gyration which is a result of both interaction (thermal) and entropic contributions and therefore more reliable as a direct measure.

**Figure 6:**
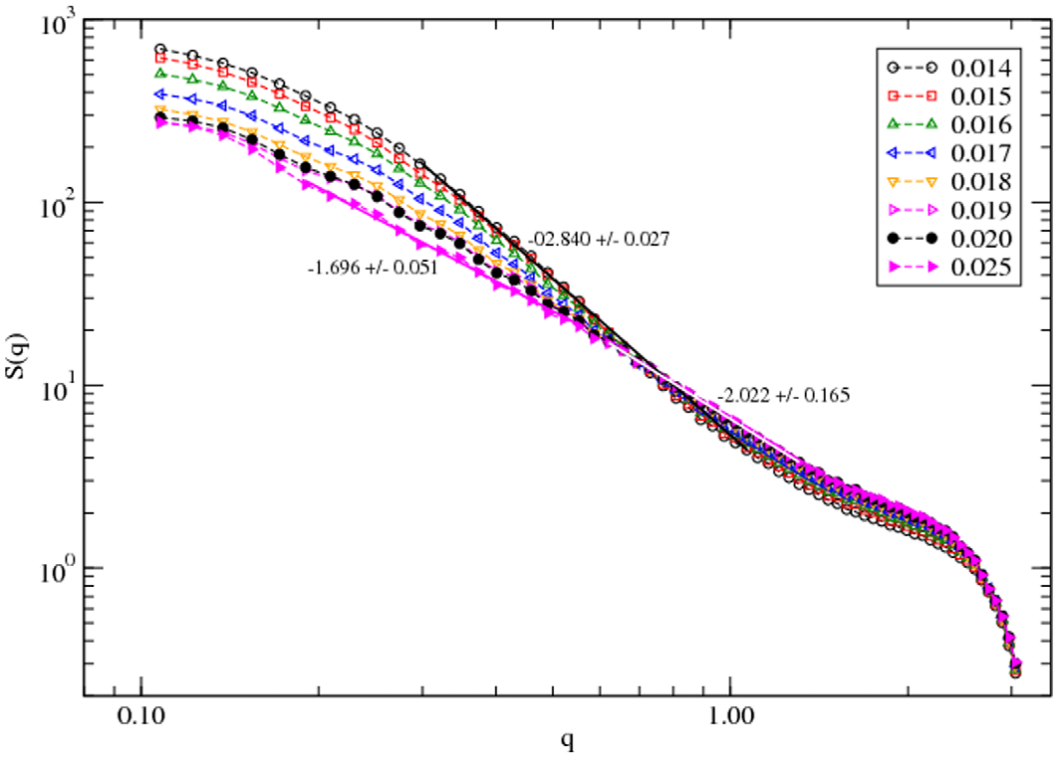
Structure factor 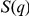 versus wave vector 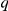. The effective dimension of the protein is 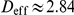 (almost solid, a globular structure) at 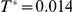 and 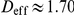 (a ramified, tenuous SAW structure) at 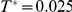.

The structure factor 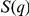 provides the spatial scaling of the distribution of constitutive elements
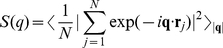
(2)where 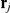 is the position vector of each residue and 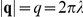 is the average spherical wave vector of wave length 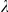. For the protein, the structure factor is useful in understanding the structural details over a range of length scales. Fig. 6 shows the variation of 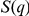 with the wave vector on a log-log scale. Since the radius of gyration 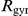 is a measure of the residue spread, the distribution of residues in the range of 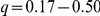 provides information about the global conformation of the protein. From the power-law scaling of the structure factor with the wave vector, 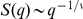, we can estimate the distribution of the protein segments 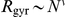. We have estimated the slope of the power-laws in the appropriate range of the wave vector 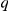 in Fig. 6: 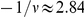 at 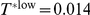 and 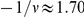 at 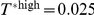 which provides 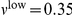 and 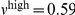, respectively. Thus, the protein has an effective dimension 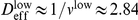 (almost solid, a globular structure) at 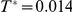 and 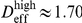 (a ramified, tenuous SAW structure) at 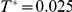. As shown in Fig. 6 a systematic change in the mass distribution of the protein is clearly seen on increasing the temperature (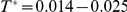). On the lower spatial scale (higher 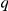) there are minor modifications in the mass distribution at 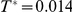 while the protein segments appear like an ideal chain with 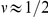 at 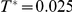.

## Discussion

In summary, a coarse-grained protein model is used to study the structural dynamics of the histone H2AX which consists of 143 residues tethered in a bond fluctuating chain on a cubic lattice. Although the atomistic details of residues are ignored, their specificity is captured via a knowledge-based interaction matrix as well as a LJ pair potential for residue-residue interactions. Each residue executes its stochastic motion according to the Metropolis criterion.

We have analyzed a number of local and global physical quantities such as the energy and mobility of each residue as well as the root mean squared displacement of the protein's center of mass, its radius of gyration, and its structure factor. The impact of temperature on these quantities is investigated and might be exploited for the design of biomaterials.

Our approach allows for the identification of segmental characteristics such as active regions and anchoring sites of the protein. We find that the electrostatic residues (e.g. 72R, 73D, 91D, 92E, 93E, 134K, 135K, etc.) are critical in orchestrating the segmental conformation, their self-assembly and de-segregation from the low to the moderately high temperature regime (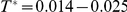). These highly interacting residues at their specific positions in the protein sequence appear to determine specificity and multi-scale structures. Accordingly, we observe global globular configurations at low (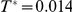), a combination of chains segments and smaller segmental globules at intermediate (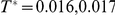), and elongated structures at moderately high temperatures (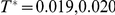). As expected, the specificity of residues vanishes at high temperatures (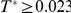) where the mobility of most residues becomes considerably high and comparable. In this thermal-driven structural regime, the residues become indistinguishable leading to a SAW chain conformation.

The radius of gyration of the protein shows a non-monotonic dependence on the temperature with a maximum at a characteristic temperature which is determined by the competition between inter-residue interactions and temperature. The protein H2AX expands (positive thermal response) on heating in the low temperature regime (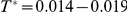) and contracts (negative thermal response) at higher temperatures (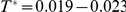). The crossover from a positive to negative thermal response occurs at a well-defined transition temperature (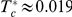) which may be a specific characteristic of the histone H2AX and particularly interesting for bioengineering purposes. The variation in the global conformation of the protein is explained in the framework of self-assembly at the local scale.

Based on the analysis of the structure factor 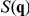, we find that the radius of gyration scales with its molecular weight 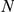 as 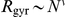, where 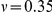 and 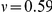 at 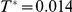 and 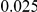, respectively. The effective dimension of the protein is therefore 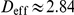 (almost solid, a globular structure) at 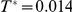 and 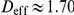 (a ramified, tenuous SAW structure) at 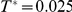. A systematic change in the mass distribution is clearly seen with an increase in temperature (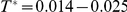).

Our coarse-grained protein model allows for a deeper understanding of local and global properties, which can so far not be gained by experimental testing. Besides the biological importance of proteins such as H2AX, we are able to provide a framework for analyzing potential candidates for the bioengineering of nano-materials. To this end, future experiments measuring physical quantities such as the spatial extension (radius of gyration) as a function of temperature would allow for the calibration of the temperature scale.
